# Ensemble perception without phenomenal awareness of elements

**DOI:** 10.1038/s41598-022-15850-y

**Published:** 2022-07-13

**Authors:** Taisei Sekimoto, Isamu Motoyoshi

**Affiliations:** Department of Life Sciences, The University of Tokyo, Tokyo, Japan

**Keywords:** Human behaviour, Sensory processing, Visual system

## Abstract

Humans efficiently recognize complex scenes by grouping multiple features and objects into ensembles. It has been suggested that ensemble processing does not require, or even impairs, conscious discrimination of individual element properties. The present study examined whether ensemble perception requires phenomenal awareness of elements. We asked observers to judge the mean orientation of a line-based texture pattern whose central region was made invisible by backward masks. Masks were composed of either a Mondrian pattern (Exp. 1) or of an annular contour (Exp. 2) which, unlike the Mondrian, did not overlap spatially with elements in the central region. In the Mondrian-mask experiment, perceived mean orientation was determined only by visible elements outside the central region. However, in the annular-mask experiment, perceived mean orientation matched the mean orientation of all elements, including invisible elements within the central region. Results suggest that the visual system can compute spatial ensembles even without phenomenal awareness of stimuli.

## Introduction

The human visual system can easily and rapidly recognize objects, scenes, and materials from complex natural images^1–3^. In order to achieve such efficient information processing, the visual system is thought to represent the statistical—or “collective”—aspects of spatially distributed information as ensembles^4–6^. Indeed, increasing psychophysical evidence shows that humans can perceive not only ensembles of low-level image features such as orientation and size^4,7^ but also ensembles of high-level objects such as faces, biological motion, etc.^8–11^. Moreover, another line of evidence points out that ensemble coding can explain visual phenomena such as search and crowding^12–16^. In the cognitive-processing literature, ensemble representations have been proposed as helpful compact codes for handling large numbers of features and objects in working-memory systems with highly limited capacities^17^.

A defining characteristic of ensemble perception is that it does not require conscious access to individual elements^6^. A number of studies suggest that human observers can accurately discriminate averages and other statistics across stimuli without conscious access to individual element properties (e.g. orientation)^8,9,18,19^. On the other hand, if a stimulus is perceived as an ensemble, it becomes difficult to discriminate the properties of its constituent elements at any given location, especially in the peripheral visual field^20–23^. This 'crowding' phenomenon has been investigated extensively, and it is generally believed to result from the impairment of conscious access to the spatial location or properties of the target elements^21–23^. In this respect, ensemble perception is inextricably linked to crowding^24^.

In a nutshell, ensemble perception does not require 'access' awareness of stimulus elements. But it is unclear whether ensemble perception requires 'phenomenal' awareness of stimulus elements^25^. Many psychophysical studies have introduced visual displays such as masking, adaptation, and binocular rivalry^26–30^, in which human observers are not only unable to access stimulus properties but are also unaware of the presence of the stimulus itself (i.e., the stimulus is phenomenally 'invisible'), despite implicit underlying neural processing of the stimulus^31–35^. In such displays, do human observers can correctly perceive an ensemble (e.g., spatial average) if a portion of the stimulus' elements are invisible?

Given vision's functional requirement that vast amounts of information be processed instantaneously, it is plausible to assume that ensemble perception does not require phenomenal awareness of elements. Alternatively, it has been shown that phenomenal awareness of elements is necessary for crowding, a percept closely related to ensemble perception. Wallis and Bex (2011), for instance, showed that crowding does not occur if a proportion of elements are made invisible by adaptation^36^ (but see also^37^). This result would seem to imply that phenomenal awareness of elements is in fact necessary for ensemble perception.

To elucidate the role of phenomenal awareness in ensemble perception, we examined whether perceived mean orientation of line-segment textures is affected by elements made invisible via dichoptic backward masking. In Experiment 1, we briefly presented a texture pattern whose central region was tilted relative to the surrounding region while the central region was backward-masked with a Mondrian pattern. Observers were asked to estimate mean texture orientation as a whole. With the central texture region almost totally invisible, observers reported mean orientations close to those of visible elements in the surrounding region. In Experiment 2, we used an annular contour mask that was designed to make central elements invisible without spatially overlapping with them. We found that the central region was perceived as a uniform hole with no visible elements, but the estimated mean orientation was consistent with the mean orientation of all elements including the invisible elements in the central region. These results suggest that, at least in the perception of average orientation, the visual system can compute spatial ensembles without phenomenal awareness of elements.

## Experiment 1

### Methods

#### Observers

We initially recruited 9 participants whose results showed that the expected effect—an interaction between the mask condition and the center orientation—was statistically significant. Based on the effect size in this result, we conducted an a priori power analysis using G*Power 3.1 which revealed that a sample size of 10 should be sufficient to achieve 0.90 power^38^. We then excluded 1 author and recruited 2 more participants, and finally had 10 naïve students participated in the experiment (3 females, 7 males, 21.8 years old on average). All observers had normal or corrected vision. All experiments were conducted in accordance with the Declaration of Helsinki and with the permission of the Ethical Review Committee for Experimental Research on Human Subjects, Graduate School of Arts and Sciences, the University of Tokyo. All observers provided filled informed consent forms.

#### Apparatus

Visual stimuli were displayed on an LCD monitor (BenQ XL2735-B) with a frame rate of 60 Hz and a pixel resolution of 0.022 deg/pixel. The luminance of the monitor was gamma-corrected based on careful measurements with a colorimeter (ColorCal II CRS). In line with previous studies^39,40^, all stimuli were presented dichoptically through a mirror stereoscope on two backgrounds (16(W) * 16(H) deg) displayed on the left and right sides of the monitor. The dichoptic presentation was effective to prevent low-level monocular (eg, retinal) interference^28^. To facilitate binocular fusion, both backgrounds were surrounded by thin frames made of random dots.

#### Stimuli

**Figure 1:**
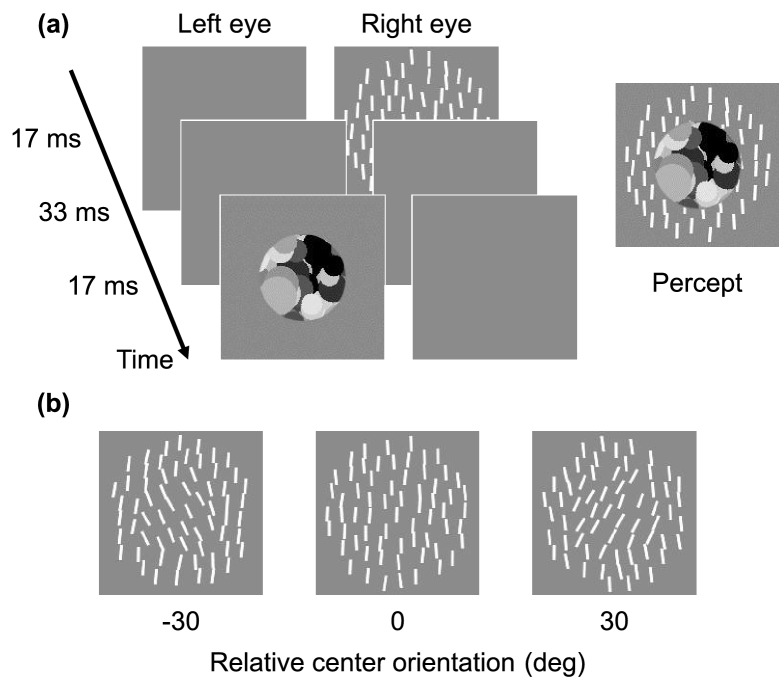
Stimuli used in Experiment 1. (**a**) Schematic of the temporal sequence of the stimuli. A line-based texture was briefly shown followed by a Mondrian pattern mask with an SOA. The right image illustrates a typical percept of the display. (**b**) The central region of the texture was varied by − 30, 0, and + 30 deg relative to the surrounding region.

Target stimuli were texture patterns consisting of 64 line segments scattered in a circular area of 3.7 deg in diameter (Fig. 1). Each line segment had a width of 0.1 deg, a length of 0.4 deg, and a luminance of 30 cd/m^2^. The elements were randomly arranged with the minimum distance of 0.3 deg. Element orientation was determined according to a Gaussian distribution with a particular mean and a s.d. of 4 deg. Elements within the circular central region of a 2.0 deg diameter were varied by − 30, 0, or 30 deg with respect to elements in the surrounding region. The mean orientation of the entire texture was determined for each trial in accordance with a staircase procedure. The mask was a circular Mondrian pattern (2.0 deg diameter) consisting of overlaid discs of random sizes and luminances. In order to check whether the observer properly reported the visibility of the central region, a texture with no central region (i.e. holed) was presented in 1 out of 5 trials.

#### Procedure

Perceived mean texture orientation was measured with a two-alternative forced-choice (2AFC) method. In each trial, the target texture was presented to either eye for 17 ms, and the mask was presented to the other eye for 17 ms following a 50 ms SOA. The target and mask were presented 4.9 deg to the right of a black fixation point (0.2 × 0.2 deg) shown continuously in the center of the background. No mask was presented in half the trials. While viewing the display with steady fixation, observers indicated by the press a button whether the mean orientation of the whole texture was tilted clockwise or counter-clockwise. Observers then pressed a button to indicate whether or not they perceived texture elements in the central region. Observers were instructed to answer “perceived” if they could perceive any line segments in the central region. The next trial was started approximately 0.5 s after the observer's response.

In accordance with the staircase method, mean texture orientation was varied every 2 deg depending on the observer's response. In each session, multiple staircase sequences corresponding to different experimental conditions were randomly interleaved. For each observer, at least 250 trials of data were collected for each central-orientation condition. The mean orientation of elements in the surrounding region giving a 50% clockwise response rate, defined as the subjective point of equivalence (PSE), was estimated via logit analysis and maximum likelihood^41,42^. Trials in which masking did not occur were not used in the estimation of PSE in the masked condition. In order to cancel any bias in the perception of absolute vertical orientation for each observer, the PSE shift was calculated by subtracting the PSE at a relative central orientation of 0 deg from the PSE obtained for each condition.

### Results

**Figure 2:**
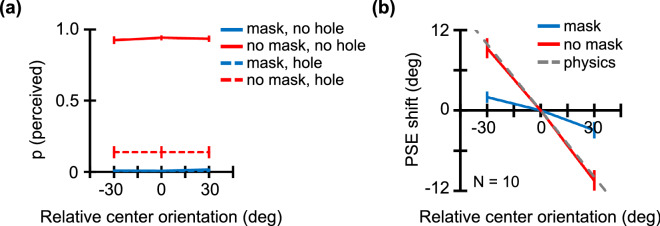
Results for Experiment 1. The red line shows results for the no-mask condition, and the blue line shows results for the masked condition. (**a**) The proportion of trials in which any line segments were perceived in the central region as a function of the relative orientation of the central region. The dashed line represents results for the texture with no central region. (**b**) The PSE shift as a function of the central region's relative orientation. The gray dashed line denotes the physical mean orientation of all elements. Error bars represent ±1 SEM.

Figure 2a shows the proportion of trials in which observers perceived any line segments in the central region. In the no-mask condition (red), observers correctly discriminated between the physically filled and holed textures. In the masked condition (blue), observers rarely perceived the filled texture in the central region, thereby indicating a robust masking effect. Figure 2b shows the PSE shift as a function of the relative orientation of the central region. In the no-mask condition (red), the PSE shift is fairly close to the physical mean orientation of all elements as denoted by the gray dashed line. Alternatively, in the masked condition (blue), the PSE shift is close to zero regardless of the central region's relative orientation.

A two-way repeated measures ANOVA was performed with mask condition and relative center orientation as factors. To improve the estimate of the probability of obtaining the results, we report the key interaction for both the initial and full samples, as well as the *p*_augmented_ statistic^43^. After running the first 9 participants, results showed significant main effects of the relative center orientation (F(1, 32) = 80.2, *p* < 0.0001, η^2^ = 0.56, 1-β = 0.99), an interaction between the two factors (F(1, 32) = 30.2, *p* < 0.0001, η^2^ = 0.21, 1-β = 0.89), and no significant main effects of the mask (F(1, 32) = 0.11, *p* = 0.74, η^2^ < 0.01, 1-β = 0.05). For the full sample of 10 participants, results showed significant main effects of the relative center orientation (F(1, 36) = 88, *p* < 0.0001, η^2^ = 0.57, 1-β = 0.99, *p*_augmented_ = [0.01, 0.01]), an interaction between the two factors (F(1, 36) = 31, *p* < 0.0001, η^2^ = 0.20, 1-β = 0.90, *p*_augmented_ = [0.01, 0.01]), and no significant main effects of the mask (F(1, 36) = 0.008, *p* = 0.93, η^2^ < 0.01, 1-β = 0.05, *p*_augmented_ = [0.87, 0.93]). In the no-mask condition, the PSE is significantly shifted for both the − 30 deg (two-tailed paired t test; t(9) = 6.26, *p* = 0.0002, d = 1.98, 1-β = 0.98) and 30 deg (t(9) = − 6.56, *p* = 0.0001, d = 2.07, 1-β = 0.99) relative orientations of the central region. In the masked condition, on the other hand, the PSE is not significantly shifted for both the − 30 deg (two-tailed paired t test; t(9) = 2.34, *p* = 0.04, d = 0.74, 1-β = 0.17) and 30 deg (t(9) = − 2.41, *p* = 0.04, d = 0.76, 1-β = 0.19) relative orientations of the central region. Furthermore, between the mask and no-mask conditions, the PSE shift is significantly different for both the − 30 deg (two-tailed paired t test; t(9) = − 4.55, *p* = 0.001, d = 2.00, 1-β = 0.99) and 30 deg (t(9) = 4.79, *p* = 0.001, d = 1.68, 1-β = 0.99) relative orientations of the central region.

In summary, our data indicate that observers estimated the mean orientation of the texture similar to those of visible elements in the surrounding region. Results also support the conclusion that observers discounted the orientation of elements in the central region made invisible by the Mondrian mask. When presented with a texture with no central region (i.e. holed), observers reported ‘perceived’ on trials with a probability of 13% even in the no mask condition. This is likely due to the strong instructions which encouraged observers to respond whenever they saw anything other than a mask in the central regions.

## Experiment 2

The results of Experiment 1 suggest that ensemble perception depends on the phenomenal awareness of masked elements. However, this conclusion discounts the possibility that orientation information in the central region was compromised by spatially overlapping luminance / pattern signals in the Mondrian mask. For example, edges in the Mondrian could have suppressed local orientation codes for individual elements. Even if we allow that neural representation of such low-level image features could survive under backward-masking conditions^31–35^, it is still possible that information in texture and mask confounded the formation of ensemble representations. Therefore, in Experiment 2, we used a circular contour mask that prevented such spatial interference. Indeed, it is known that circular-contour masks strongly suppress conscious texture perception inside the annulus^39^ but preserves the neural processing responsible for texture segregation and pop-out^40^.

### Method

**Figure 3:**
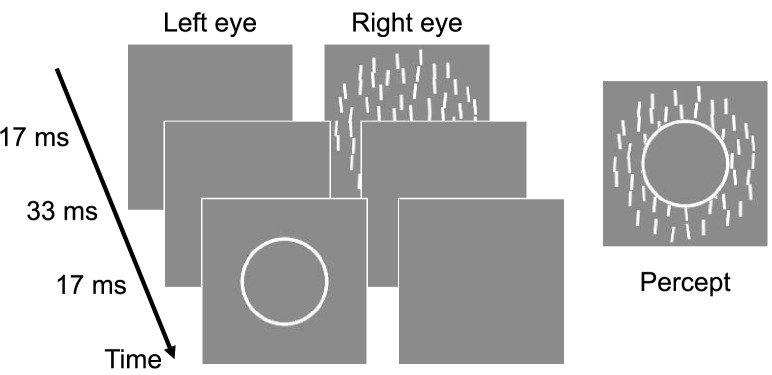
Stimuli used in Experiment 2. Schematic of the temporal sequence of the stimuli. A line-based texture was briefly shown followed by an annular contour mask with an SOA. The right image illustrates a typical percept of the display.

In this experiment, the mask in Experiment 1 was replaced by a circular contour with a diameter of 2.0 deg (Fig. 3). The annulus consisted of a thin contour with a width of 0.2 deg and a luminance of 35 cd/m^2^. To ensure that there was no phenomenal awareness, observers were given strict instructions to respond “perceived” if they saw anything other than a uniform hole in the central region of the texture. The others were the same as in Experiment 1. We initially recruited 9 participants, but two out of nine who could not discriminate texture orientation at all were excluded from the analysis. Based on sample size of observers in Experiment 1, we added four more observers to guard against power loss due to planned data exclusions referring to the early stage of the experiment. In the end, thirteen observers, including twelve naïve students and one of the authors, participated in the experiment, but three observers were excluded from the analysis.

### Results

**Figure 4:**
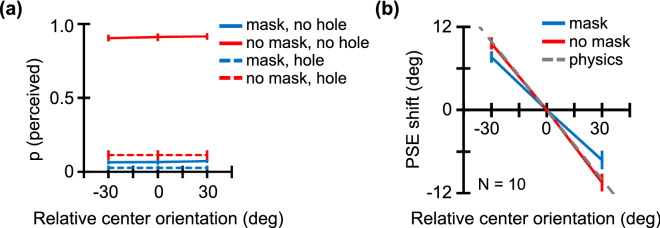
Results for Experiment 2. The red line shows results for the no-mask condition, and the blue line shows results for the masked condition. (**a**) The proportion of trials for which anything other than a uniform hole was perceived in the central region as a function of the central region's relative orientation. The dashed line represents results for the texture with no central region. (**b**) The PSE shift as a function of the central region's relative orientation. The gray dashed line denotes the mean physical orientation of all elements. Error bars represent ±1 SEM.

Figure 4a shows the proportion of trials in which anything other than a uniform hole was perceived in the central region. We found that the texture in the central region (i.e., the one corresponding to the inside of the annular mask) was invisible in almost 100% of the trials in the masked condition. Figure 4b shows the PSE shift as a function of the central region's relative mean orientation. In contrast to Experiment 1, the PSE in both the no-mask (red) and mask (blue) conditions was shifted remarkably close to the physical mean of all elements, including the invisible elements in the central region.

A two-way repeated measures ANOVA was performed with mask condition and relative center orientation as factors. After running the first 7 participants, results showed a significant main effect of relative center orientation (F(1, 24) = 196, *p* < 0.0001, η^2^ = 0.86, 1-β = 0.99), a non-significant main effect of mask condition (F(1, 24) = 1.20, *p* = 0.29, η^2^ = 0.01, 1-β = 0.06), and a weak interaction between the two factors (F(1, 24) = 5.89, *p* = 0.02, η^2^ = 0.03, 1-β = 0.18). For the full sample of 10 participants, results showed a significant main effect of relative center orientation (F(1, 36) = 246, *p* < 0.0001, η^2^ = 0.86, 1-β = 0.99, *p*_augmented_ = [0.01, 0.01]), a non-significant main effect of mask condition (F(1, 36) = 0.34, *p* = 0.57, η^2^ < 0.01, 1-β = 0.05, *p*_augmented_ = [0.41, 0.57]), and a weak interaction between the two factors (F(1, 36) = 5.49, *p* = 0.02, η^2^ = 0.02, 1-β = 0.13, *p*_augmented_ = [0.01, 0.03]). In the no-mask condition, the PSE is significantly shifted for both the − 30 deg (two-tailed paired t test; t(9) = 10.9, *p* < 0.0001, d = 3.45, 1-β = 0.99) and 30 deg (t(9) = − 8.16, *p* < 0.0001, d = 2.58, 1-β = 0.99) relative orientations of the central region. Similarly, in the masked condition, the PSE is shifted significantly in both the central region's − 30 deg (t(9) = 9.20, *p* < 0.0001, d = 2.91, 1-β = 0.99) and 30 deg (t(9) = − 5.30, *p* = 0.0005, d = 1.67, 1-β = 0.90) relative-orientation conditions.

In summary, results of Experiment 2 indicate that observers assessed mean texture orientation using all elements, even without phenomenal awareness of elements in the central region made invisible by the annular mask. Our data support the notion that ensembles are readily computed without conscious detection of elements, provided that there is no interference from other stimuli such as the mask used in Experiment 1. In the no mask condition, observers still reported 'perceived' in 11% of trials in which textures had no central region (i.e. holed). This finding is probably attributable to our strict instruction to respond “perceived” whenever the observers saw anything other than a uniform hole in the central region.

## General discussions

The present paper has sought to clarify the role of phenomenal awareness of texture elements in ensemble perception. To this end, we used a backward masking paradigm to measure whether mean perceived orientation of line-based textures is affected by invisible elements. A first experiment with a Mondrian mask suggested that perceived mean orientation was computed mainly from visible elements. However, a second experiment with a spatially non-overlapping annular contour mask revealed that observers computed mean orientation using all elements, including those elements made invisible by masking. These results suggest that human vision does not necessarily require phenomenal awareness of elements to spatially average the orientation of an ensemble.

These results are in favor of the idea that the visual system can efficiently recognize and memorize complex scenes by representing multiple elements of information as ensembles^5,6^. In order to achieve such tiered functionality, the visual system must rapidly compute ensembles before the stimulus reaches conscious visual awareness. Indeed, there is much evidence that textures composed of low-level image features, such as orientation, are processed implicitly in the early visual cortex such as V1 and V2^44–47^. It has also been suggested that conscious awareness of visual stimuli is mediated by feedback loops from higher-order visual areas^48–50^, and these feedback signals are known to be suppressed by backward masking or transcranial magnetic stimulation (TMS) applied slightly later than stimulus onset^32,51^. According to these findings, visual cortex could generate ensemble representations in the feed-forward process prior to recurrent inputs from the feedback loop. Presumably, it is this feedback loop that our backward-masking display managed to disrupt.

Our data appear inconsistent with previous findings of crowding—a percept closely related to ensemble perception—in that ensemble perception does not always require phenomenal awareness whereas crowding is strongly correlated to phenomenal awareness^36^ (but see also^37^). However, crowding is defined as the difficulty in discriminating the property of a specific local element in a set (i.e. a problem of access)^52^. Reconciling apparent discrepancies between our results and the crowding literature may therefore hinge on the involvement of higher-order attentive processes rather than pre-attentive processes in ensemble computation. Indeed, crowding is often attributed to the spatial distribution of attention associated with localization errors and eye movements rather than to lower-order processes such as lateral interactions between elements^22,53,54^. In a word, the visual system may transform the visual input into ensembles or textures codes, and these representations may be computed before conscious stimulus detection. In such a functional architecture, ensemble representations would enter consciousness via feedback loops. One may hypothesize that crowding occurs because information about an element at a given location is no longer accessible in compact ensemble representations.

In our experiments, observers utilized invisible elements to estimate mean orientation in the annular-mask condition but not in the Mondrian-mask condition. We take this as evidence that luminance and pattern information inside the Mondrian mask interferes with orientation information in the central region of the texture. There are several possible interpretations as to the specific level of visual information processing at which interference occurs. One possibility is that the Mondrian mask inhibited local orientation signals of individual texture elements, but this account is inconsistent with the findings that stimulus features are encoded even if stimulus perception is lost due to masking or binocular rivalry^31–35^. Given that observers reported they could not see any line segments inside the central region, it is also unlikely that observer consciously perceived a mixture of texture and Mondrian. At the unconscious level, however, it is possible that information in the Mondrian was temporally averaged with (local and/or ensemble) representations of the central texture region. With the contour mask, on the other hand, invisible elements also contributed to the ensemble, since stimulus visibility was suppressed without any direct interference or averaging from the mask signal. Whereas the pattern mask has been widely used as a visual stimulus to impair the conscious perception of a target stimulus^28^, the results of Exp. 1 may bring a caution about the nature of mask interference on the signal^55^ when considering different classes of implicit representations.

The results of Experiment 2 imply that observers estimated mean orientation of all texture elements, including those in the masked central region. Yet, it is also true that observers achieved this judgment by seeing only the visible surrounding region. One explanation for this paradoxical observation is that observers had access to the neural representation of mean orientation of the whole texture which was formed at the unconscious level, then made a judgement independently how they perceived the visible surrounding regions. Another possible account is that observers made a judgment upon the apparent orientation of the visible surrounding regions as spatially averaged with the orientation of the masked central region. This explanation appears to be inconsistent with recent psychophysical data showing that perceptual assimilation between two clearly distinct uniform stimuli requires phenomenal awareness of the stimuli^56^. On the other hand, it has been reported that compulsory averaging of the apparent orientation of local elements occurs in textures with noisy oriented elements like our stimuli^24^. Our results could also be interpreted as indicating that such averaging, which can be regarded as partially equivalent to ensemble perception, takes place before conscious stimulus detection.

The results of the present study suggest the visual system can compute spatial ensembles without phenomenal awareness for low-level image features such as orientation. Yet, it is still unclear that the same would hold true for complex features such as faces and objects. Several psychophysical studies have pointed out that the computation of face ensembles may be a product of rapid serial encoding in a process with limited capacity^57^. It has also been suggested that the perception of temporal ensembles of serially sampled visual information involves distinct processes from the computation of spatial ensembles^58^. Importantly, though, it is known that the perception of temporal ensembles requires conscious access to the property of individual samples^59^. Therefore, it is possible that spatial ensemble perception of high-level attributes (e.g., faces) requires conscious awareness of elements. Testing this possibility will be useful for comprehensive understanding of multi-level mechanisms for ensemble perception.

## Data Availability

The datasets generated and analyzed during the current study are available from the corresponding author on reasonable request.
